# Protogenin, a new member of the immunoglobulin superfamily, is implicated in the development of the mouse lower first molar

**DOI:** 10.1186/1471-213X-10-115

**Published:** 2010-11-25

**Authors:** Keiko F Takahashi, Tamotsu Kiyoshima, Ieyoshi Kobayashi, Ming Xie, Haruyoshi Yamaza, Hiroaki Fujiwara, Yukiko Ookuma, Kengo Nagata, Hiroko Wada, Takako Sakai, Yoshihiro Terada, Hidetaka Sakai

**Affiliations:** 1 Laboratory of Oral Pathology and Medicine, Faculty of Dental Science, Kyushu University, Fukuoka 812-8582, Japan; 2 Department of Prosthodontics, School of Stomatology, Ninth People's Hospital, Shanghai Jiao Tong University, 639 Zhizaoju Road, Shanghai, 200011, China; 3 Department of Pediatric Dentistry, Faculty of Dental Science, Kyushu University, Fukuoka 812-8582, Japan; 4 Department of Fixed Prosthodontics, Faculty of Dental Science, Kyushu University, Fukuoka 812-8582, Japan

## Abstract

**Background:**

*Protogenin (Prtg) *has been identified as a gene which is highly expressed in the mouse mandible at embryonic day 10.5 (E10.5) by a cDNA subtraction method between mandibles at E10.5 and E12.0. Prtg is a new member of the deleted in colorectal carcinoma (DCC) family, which is composed of DCC, Neogenin, Punc and Nope. Although these members play an important role in the development of the embryonic central nervous system, recent research has also shed on the non-neuronal organization. However, very little is known regarding the fetal requirement of the non-neuronal organization for Prtg and how this may be associated with the tooth germ development. This study examined the functional implications of Prtg in the developing tooth germ of the mouse lower first molar.

**Results:**

Ptrg is preferentially expressed in the early stage of organogenesis. Prtg mRNA and protein were widely expressed in the mesenchymal cells in the mandible at E10.5. The oral epithelial cells were also positive for Prtg. The expression intensity of Prtg after E12.0 was markedly reduced in the mesenchymal cells of the mandible, and was restricted to the area where the tooth bud was likely to be formed. Signals were also observed in the epithelial cells of the tooth germ. Weak signals were observed in the inner enamel epithelial cells at E16.0 and E18.0. An inhibition assay using a hemagglutinating virus of Japan-liposome containing *Prtg *antisense-phosphorothioated-oligodeoxynucleotide (AS-S-ODN) in cultured mandibles at E10.5 showed a significant growth inhibition in the tooth germ. The relationship between Prtg and the odontogenesis-related genes was examined in mouse E10.5 mandible, and we verified that the Bmp-4 expression had significantly been decreased in the mouse E10.5 mandible 24 hr after treatment with Prtg AS-S-ODN.

**Conclusion:**

These results indicated that the *Prtg *might be related to the initial morphogenesis of the tooth germ leading to the differentiation of the inner enamel epithelial cells in the mouse lower first molar. A better understanding of the Prtg function might thus play a critical role in revealing a precious mechanism in tooth germ development.

## Background

The organs of vertebrates are typically composed of epithelial and mesenchymal tissues. Signaling between these two tissues governs many aspects of organogenesis, from the initiation of organ development to the terminal differentiation of organ-specific cell types. The development and differentiation of the mouse tooth germ, like many other organs, depends on such inductive interactions. A large number of genes have been proven to be related to tooth morphogenesis [1-8]. However, the precise signaling pathway which is involved in the initiation, growth, and differentiation of the tooth germ has not yet been fully elucidated. There may be additional odontogenesis-related genes that have not yet been identified. A cDNA subtraction between the mandibles of embryonic day 10.5 (E10.5) and E12.0 mice was conducted to identify genes which might be related to the tooth morphogenesis. Thirty-five of the highly expressed positive clones were obtained from the E10.5 mandible by a colony array screening. In addition, 47 of the highly expressed positive clones were also obtained from the E12.0 mandible [9]. The expression of several of those genes is closely associated with the developing tooth germ [7,8,10-12]. *Protogenin (Prtg) *[13,14], which we first designated as *Clone 15*, is one of the highly expressed genes in the mouse mandible at E10.5 [9].

*Prtg *belongs to the immunoglobulin superfamily (IgSF), which is one of the largest protein families in the mammalian genome [15,16]. This family is comprised of transmembrane and cell surface proteins and its members are characterized by immunoglobulin (Ig) domains in their extracellular regions. The IgSF members act as adhesion molecules, and can also transduce signals upon ligand stimulation. Many members of the IgSF are involved in tissue formation and morphogenesis during embryonic development [15,16]. However, thus far the functions of *Prtg *have not been elucidated.

The constituents of a subgroup of the IgSF have recently received attention because of their roles in the migration and guidance of axon growth during development of the vertebrate nervous system. One of the representative genes in this subgroup is the Deleted in Colorectal Cancer (DCC) gene, and therefore this subgroup is referred to DEAL (DCC et al.), and includes DCC, Neogenin [17], Punc [18], and Nope [19]. DCC was originally identified as a tumor suppresser gene [20], but it has been recently shown to act as a Netrin receptor for cell migration and axon guidance cues [19]. Like DCC, Neogenin is a Netrin receptor. Punc [21] and Nope are prominently expressed by differentiating neurons in the central nervous system. They are involved in the early stages of nerve tissue morphogenesis. *Prtg *belongs to DEAL because their structures are highly homologous. There are two reports in which the expression of *Prtg *was described in chick [13], mouse, and zebrafish [14]. These reports demonstrated that *Prtg *is expressed in the central nervous system in the early developmental stages of the embryo. Vesque et al. [14] demonstrated that this gene is expressed in the first branchial arch as well as in the central nervous system. This finding supported a previous study [9] in which *Prtg *was preferentially expressed in the first branchial arch prior to tooth germ formation. Therefore, it is possible that the *Prtg *gene is related to the morphogenesis of the tooth germ because the tooth germ develops under the influence of cells in the first branchial arch. This study characterized the expression pattern of *Prtg *in the developing tooth germ, and examines the possible functional implications of this gene in tooth germ morphogenesis.

## Results

### Characterization of predicted Prtg protein

**Figure 1:**
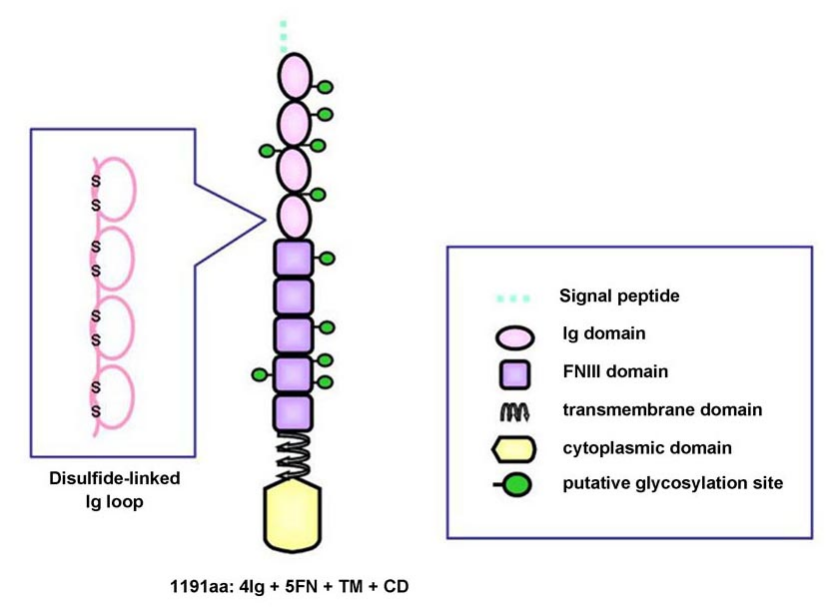
**Prtg has a characteristic domain structure and putative glycosylation sites in the extracellular region**. The Prtg protein contains a signal peptide, Ig domains, five fibronectin-type III (FNIII) repeats, a single transmembrane (TM), and a cytoplasmic domain (CD). The structure indicates a high similarity to Nope. The circles indicate glycosylated sites.

A DNA sequence analysis was performed by using of the 5'-RACE and 3'-RACE methods. Based on the DNA sequence analysis, the Prtg protein comprises 1191 amino acids, a signal peptide (SP), 4 Ig domains, 5 fibronectin (FN)-type III repeats, a single transmembrane (TM), and a cytoplasmic domain (CD). The deduced molecular structure is shown in Figure 1. In sequencing the five independent Prtg cDNA clones from E10.5 mice, no alternatively spliced variant was found within the coding region of the amino acids.

**Figure 2:**
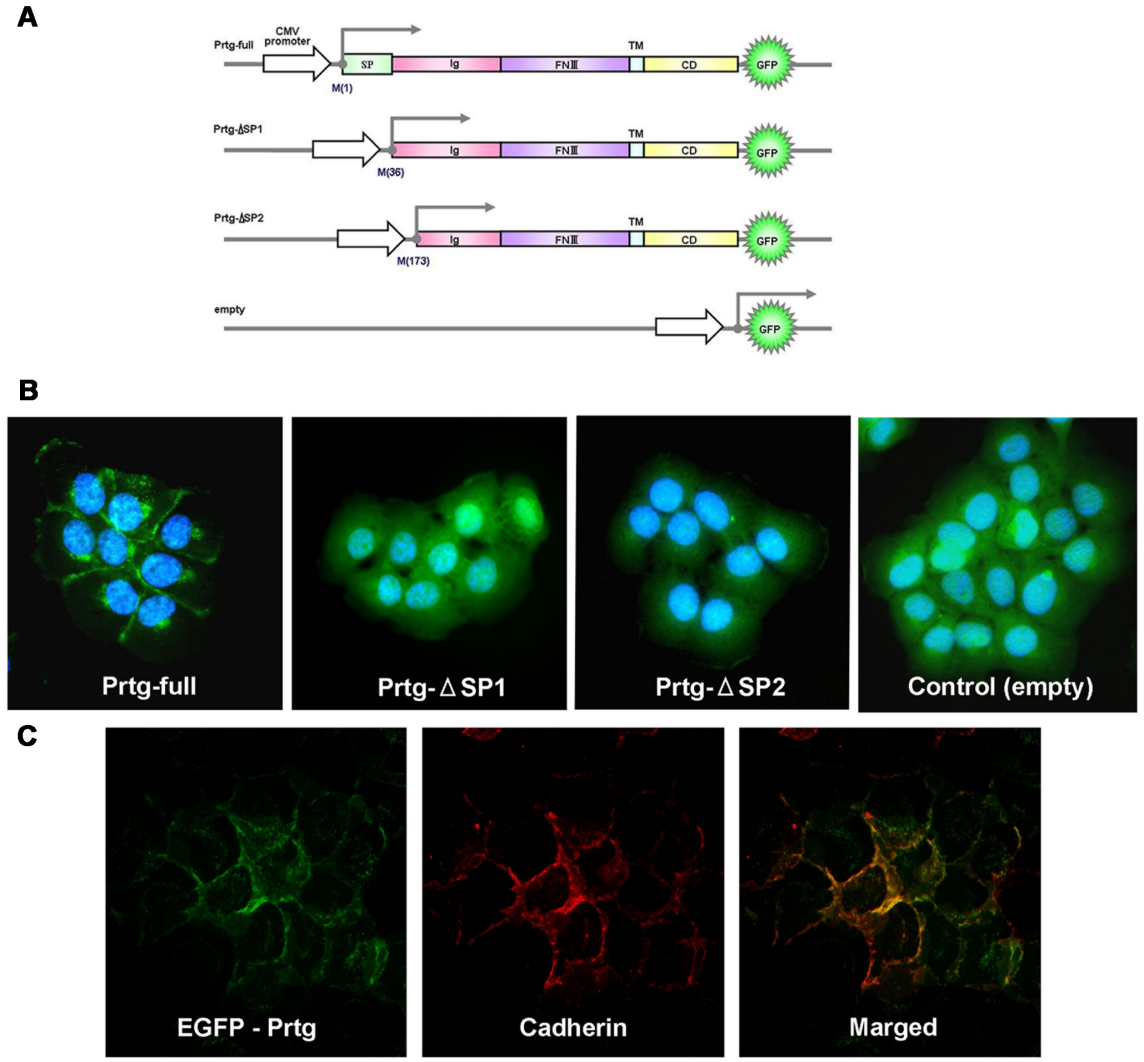
**Ptrg is a transmembrane protein**. (A) The constructs expressing various Prtg-EGFP fusion proteins are represented by a schematic drawing. The circled numbers refer to the amino acids position from methionine (M) at the initiation site of protein translation. (B) The recombinant Prtg-EGFP fusion protein is detected along the cell surface (Prtg-full), whereas the SP-deleted Prtg-EGFP fusion protein is present in pools in the cytoplasm of cells transfected with Ptrg-ΔSP1 or Prtg-ΔSP2. (C) Immunofluorescent signals for cadherin (red) overlap with the localization of the Prtg-EGFP fusion proteins in the transfected cells. The nuclei are counterstained by DAPI.

Full-length (Prtg-full) cDNA and mutant cDNA with a complete deletion of the SP region were inserted into an enhanced green fluorescent protein (EGFP) (Clontech) vector, and was then transfected into MISK81-5 cells, which is an oral squamous cell carcinoma cell line established in our laboratory [22], to characterize the intracellular localization of the Prtg protein. The MISK81-5 cells transfected with Prtg-full showed a localization of EGFP-fusion protein in the cell membrane by fluorescence microscopy (Figure 2B). Meanwhile, the other transfectants with Ptrg-ΔSP1, Prtg-ΔSP2, or empty vector showed a diffuse intracellular Prtg distribution (Figure 2B). Immunofluorescent staining for cadherin, a marker of the cell membrane-associated protein, in the transfectants with Prtg-full showed that the fluorescence images of Prtg-EGFP fusion protein and cadherin were merged (Figure 2C). These results indicated that Prtg was localized in the cell membrane.

**Figure 3:**
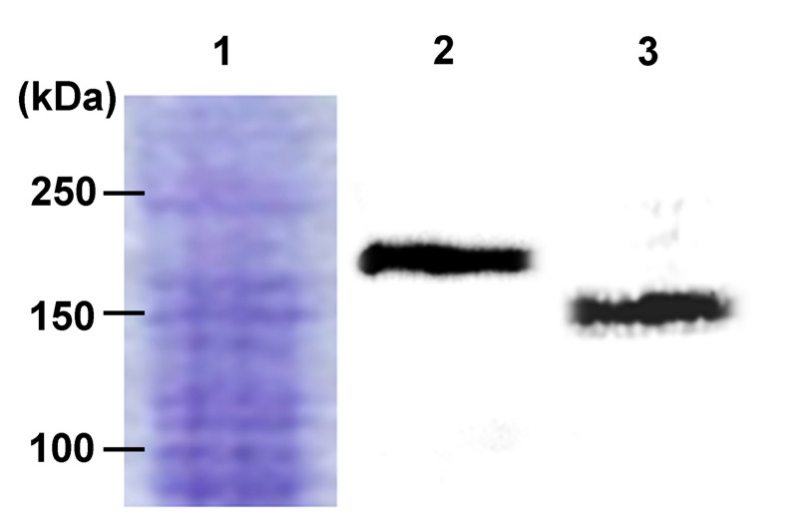
**Ptrg is a highly N-glycosylated protein**. A Western blot analysis revealed that Prtg purified from an E10.5 mandible is a protein with a molecular mass of approximately 180 kDa (lane #2). The molecular mass is much larger than that calculated from the predicted amino acid sequences. N-glycosidase F reduces the size of the proteins to approximately 130 kDa (lane #3). The purified proteins were separated and stained with Coomassie brilliant Blue dye (lane #1). The amount of the loaded protein is 10 μg in each lane.

A Western blot analysis using a Prtg affinity polyclonal anti-body demonstrated the molecular mass (*Mr*) of Prtg to be 180 kDa (Figure 3, lane 2). The mature mouse Prtg contains 1191 amino acids, and therefore the estimated *Mr *is approximately 130 kDa. Because the molecules which have SP in the extracellular domain (ECD) are often highly glycosylated, it is thought that differences in *Mr *might be caused by glycosylation at the ECD (Figure 1). Prtg protein was purified from the mandible at E10.5, and was treated with N-glycosidase F and assayed by an immunoblotting analysis to confirm ECD glycosylation and to identify the accurate size of Prtg. A Western blot analysis after the treatment of N-glycosidase F showed a reduced Prtg size (Figure 3, lane 3).

### Temporal expression analysis of Prtg mRNA and protein during odontogenesis

**Figure 4:**
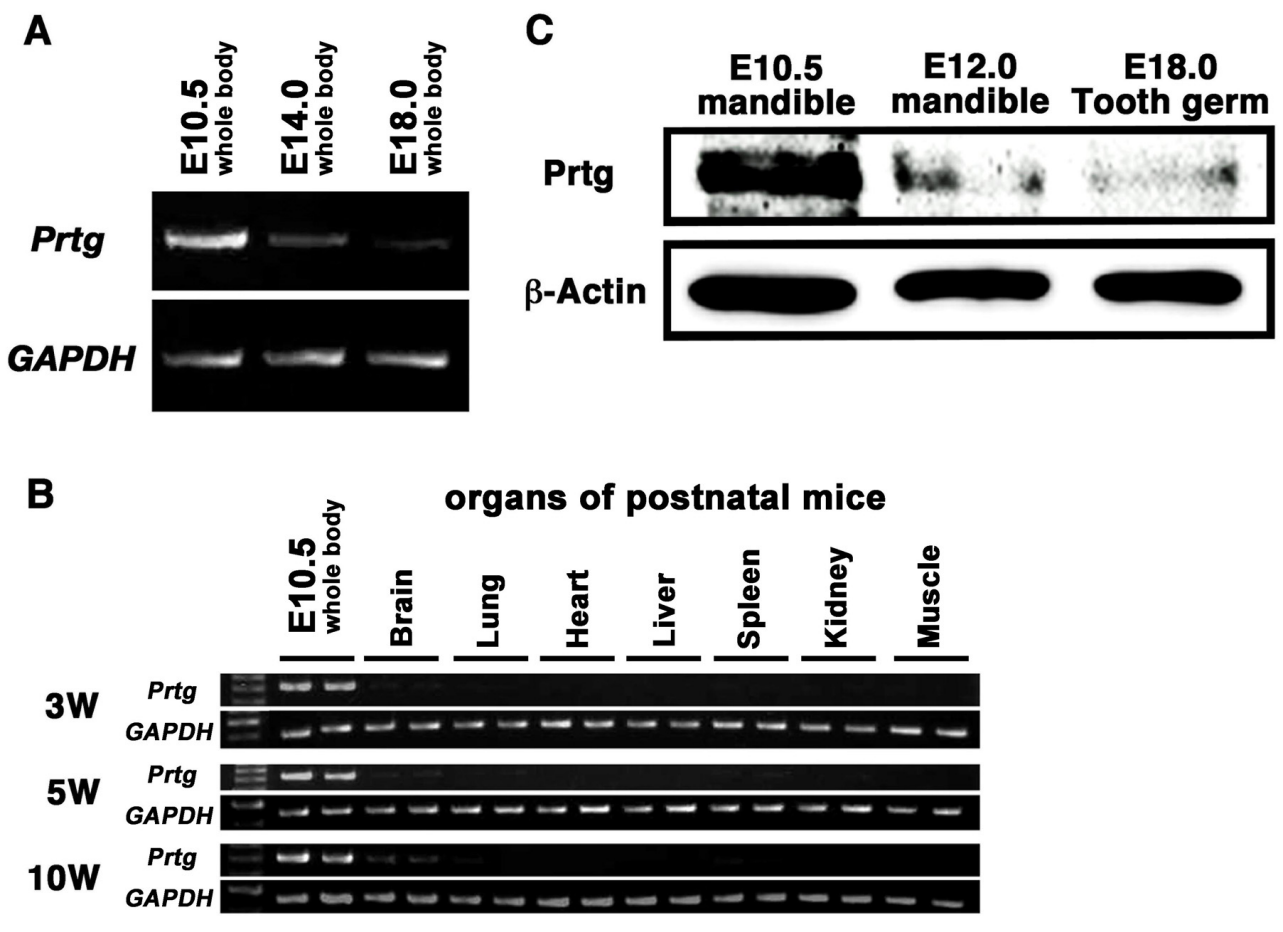
**Prtg is preferentially expressed in the early stage of organogenesis**. A semi-quantitative RT-PCR analysis of *Prtg *is compared at various embryonic stages and in various organs of postnatal mice. (A) As development proceeded from E10.5 to E14.0, the Prtg transcripts were dramatically decreased in the whole body. (B) In the postnatal mice, *Prtg *transcripts were detected only in the brain of the 10-week-old mice. However, they were remarkably reduced in comparison with that in the E10.5 whole body. Each organ group is represented by two samples. (C) According to an immunoblotting analysis, Prtg is expressed at substantially higher levels in the E10.5 mandible than in the E12.0 mandible. Prtg was dramatically decreased in the tooth germ at E18.0. The amount of the loaded protein is 10 μg in each lane.

Because *Prtg *was highly expressed in the mouse mandible at E10.5 [9], the temporal expression pattern of the Prtg mRNA during embryogenesis was examined by semi-quantitative RT-PCR with the total RNA from whole embryos. *Prtg *is highly expressed at E10.5 (Figure 4A). However, there were considerable decreases in the level of mRNA in the whole body at E14.0 and E18.0 (Figure 4A). Thereafter, the Prtg mRNA expression was examined in adult organs, and compared to the expression level of the E10.5 embryo. A weak expression was demonstrated in the central nervous system of the adult mice. Meanwhile, no expression was detected in the other organs (Figure 4B). These results indicated that Prtg mRNA is primarily expressed at the early-middle stages of embryogenesis [13,14].

The expression level of Prtg protein in the mouse mandible was also examined by a Western blotting analysis. As shown in Figure 4C, the Prtg protein levels in the mandible were higher at E10.5 than that at E12.0. The Prtg mRNA levels also decrease in the E12.0 mandible compared to the E10.5 mandible in the cDNA subtraction analysis [9]. The expression level of the Prtg protein was dramatically reduced in the tooth germ at E18.0 (Figure 4C).

### Expression of the Prtg mRNA and protein in the developing tooth germ and other organs

An *in situ *hybridization analysis was performed by using a *Prtg *antisense cRNA probe to examine the temporal and spatial expression pattern of the Prtg mRNA in the course of the developing mouse embryonal organs. *In situ *hybridization (ISH) for Prtg mRNA showed diverse signal intensity within the same tissue section. Therefore, the terms ''strong'' and ''weak'' were used only for the relative evaluation of the signal intensity in the same section.

**Figure 5:**
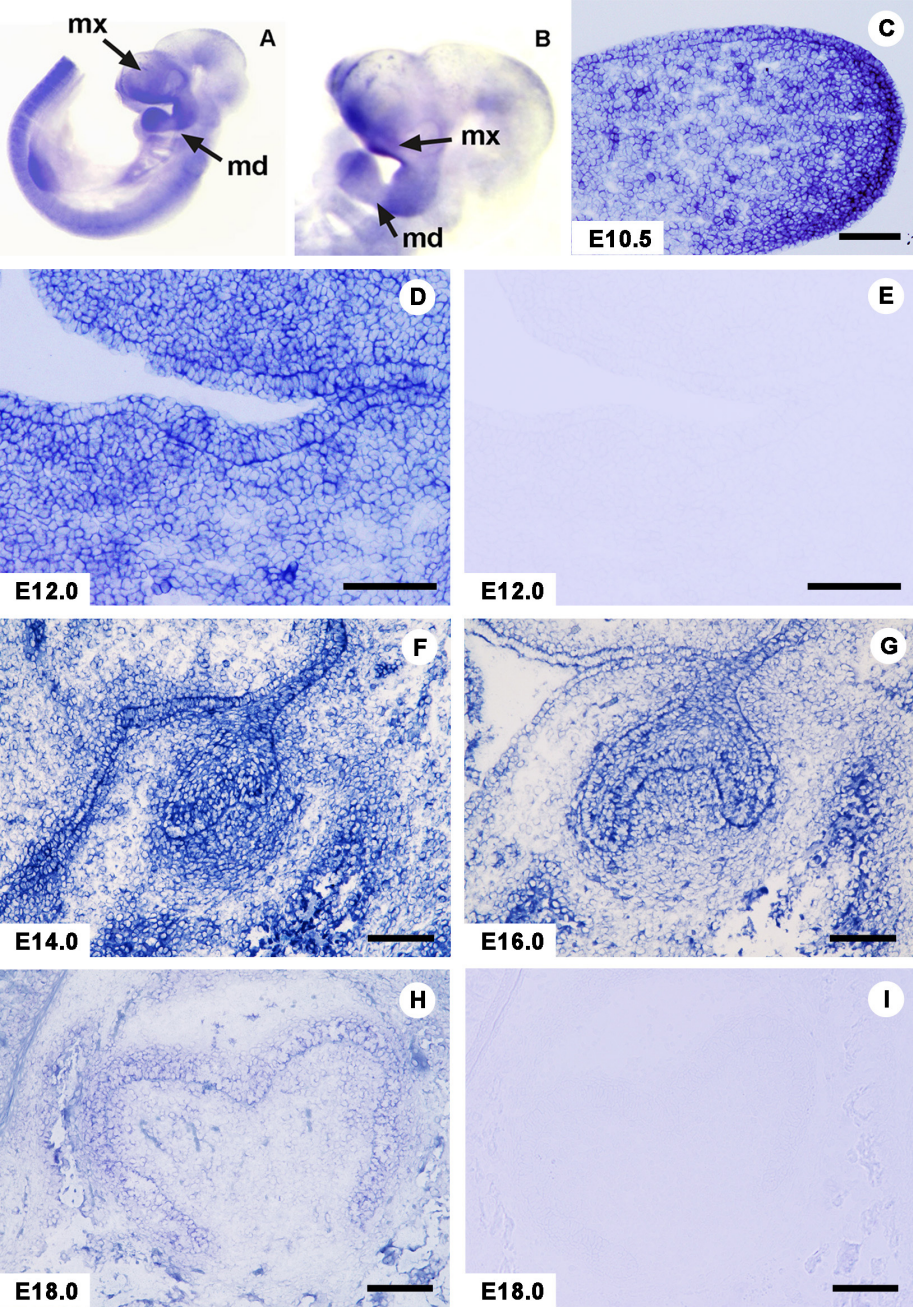
**In situ signal of Prtg mRNA is noted in the developing tooth germ.(A)** The whole mount in situ expression in an E10.5 embryo reveals that Prtg mRNA is present in the maxilla and mandible as well as in the central nervous system. **(B)** A higher magnification of Fig. 5A; mx, maxilla; md, mandible. **(C)** At E10.5, strong in situ Prtg signals were present in the mesenchymal cells in the first branchial arch and throughout the oral epithelial layer (OPL).** (D) **At E12.0, an in situ signal is observed in the OPL and underlying mesenchymal cells. **(E)** Control section with sense probe to Fig. 5D. **(F) **At E14.0, the Prtg mRNA is conspicuous in the enamel organ and the surrounding mesenchymal cells. **(G)** At E16.0, in situ signals are detected in the enamel organ and the dental papilla. **(H)** At E18.0, a faint mRNA signal is marginally detected in the inner enamel epithelium. In the dental papilla, the positive cells are noted in the presumptive caspal areas. The signal is undetectable in the other areas. **(I) **Control section with sense probe in an E18.0 tooth germ. The left side of Figs. 5C-H corresponds to the lingual side, and the right side is the buccal side. Scale bars: C- H, 200 μm.

The whole mount *in situ *expression of the Prtg mRNA at E10.5 revealed that this signal was present in the maxilla and mandible as well as central nervous system and eye, and thus the expression pattern of Prtg mRNA seemed to correspond to the distribution of the arch ectodermal cells (Figures 5A and 5B). This appeared to be similar to the results of the study by Vesque et al. [14]. In addition, in the study by Chai et al. [23], the Prtg function appeared to be involved in the cranial neural crest cells. At E10.5, a strong expression of *in situ *signal of *Prtg *was seen in the mesenchymal cells which were widely distributed in the first branchial arch including the developing mandible (Figure 5C). A signal was also found throughout the oral epithelial layer. At E12.0, *in situ *signal was observed in the oral epithelial layer, including the thickened area and underlying mesenchymal cells (Figure 5D). At E14.0, the Prtg mRNA signal was rather restricted to the enamel organ and the dental mesenchyme (Figure 5F). At E16.0, *in situ *signal of Prtg was detected in the enamel organ, in the dental papilla and in the dental sac (Figure 5G), but the intensity appeared to be reduced. At E18.0, a faint *in situ *signal of Prtg was found in the inner enamel epithelium. The faintly positive cells were localized in the presumptive cuspal areas. Weak mRNA expression was also observed in the outer enamel epithelium. However, the *in situ *signal was markedly reduced in the dental papilla (Figure 5H). A *Prtg *sense probe was applied to the tissue specimens as a control. However, no hybridization signal was detected (Figures 5E and 5I).

**Figure 6:**
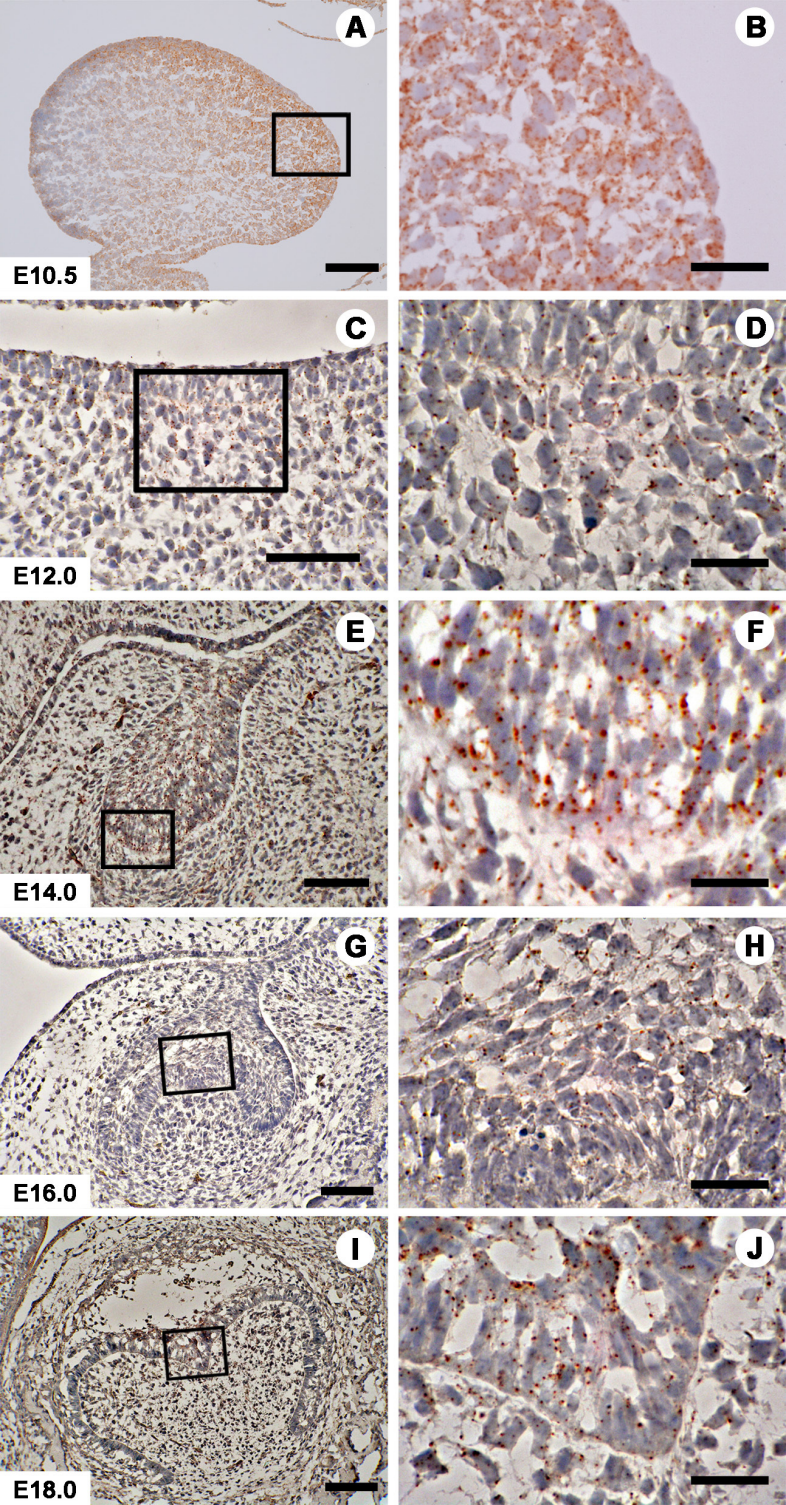
***Prtg *knockdown induces arrest of the tooth germ development.** (**A**) Prtg transcripts were dramatically decreased in the E10.5 mandible in a time-dependent manner in the organ culture (** p < 0.01). (**B**) Prtg expression in the E12.0 mandible was reduced to less than 5% of that in the E10.5 mandible (** p < 0.01). Tooth germ in the control sample or in the mandible treated with SE-S-ODN (**C**) developed to the cap stage on the 8th day of cultivation, while (**D**) an arrest of the tooth germ development was observed in samples treated with the AS-S-ODN. Immunohistochemically, Ki67-positive cells were seen in the control samples and in the mandibles treated with SE-S-ODN (**E**), and in samples treated with AS-S-ODN (**F**) on the 8th day after cultivation. (**G**) There were no significant differences in the cell proliferation in the objective areas among the control samples, the samples treated with AS-S-ODN and the samples treated with SE-S-ODN. Significant differences in the cell proliferation were noted between the DE and SM in the control samples and in the mandible treated with SE-S-ODN, while this was not observed among the areas in samples treated with AS-S-ODN (*p < 0.05, ** p < 0.01). The left sides of Figures 6C, D, E and F correspond to the buccal side, while the right side corresponds to the lingual side. Scale bars: C, D, E and F, 150 &#956m. Ut; untreated control sample, AS; samples treated with AS-S-ODN, SE; samples treated with SE-S-ODN. DE; dental epithelium, DM; dental mesenchyme, SM; surrounding mesenchyme (SM).

An immunohistochemical analysis (IHC) was also carried out using an anti-Prtg antibody. Both the protein expression and gene expression were detected in the first branchial arch in a widespread pattern, in both the epithelium and mesenchyme. Strong signals were noted near the oral epithelial layer (Figure 6A). A higher magnification showed the immunohistochemical signal of the Prtg protein to be observed surrounding the cells with a punctate appearance (Figure 6B), suggesting protein localization on the cell surface. This staining pattern was common in all the sections of each embryonic day. Although immunolocalization of the protein was present in both the epithelium and mesenchyme at E12.0, the signal intensity was reduced in comparison to that at E10.5 (Figures 6C and 6D). At E14.0, the Prtg protein signal was conspicuously detected in the enamel organ and the surrounding condensed mesenchymal cells (Figures 6E and 6F). The signal in the epithelium was stronger than that in the mesenchyme. At E16.0, immunohistochemical signal of Prtg were marginally detected in the enamel organ and in the dental papilla (Figures 6G and 6H). At E18.0, a faint immunohistochemical signal was detected in the inner and outer enamel epithelia, and in the dental papilla (Figures 6I and 6J).

Thus, both the mRNA and protein of Prtg demonstrated similar expression patterns during odontogenesis. In addition, the expression of Prtg is localized in the developing nervous system, especially in the neural tube, the retina, the lens, and the brain throughout the embryonic period.

### Functional analysis of Prtg during development of the tooth germ

The results of the *in situ *hybridization and immunohistochemical analyses suggested that *Prtg *might be involved in tooth morphogenesis. Therefore, an inhibition assay for the translation of Prtg mRNA was performed using Prtg antisense-phosphorothioated-oligodeoxynucleotide (AS-S-ODN) according to the same experimental design in our previous studies [6,7].

**Figure 7:**
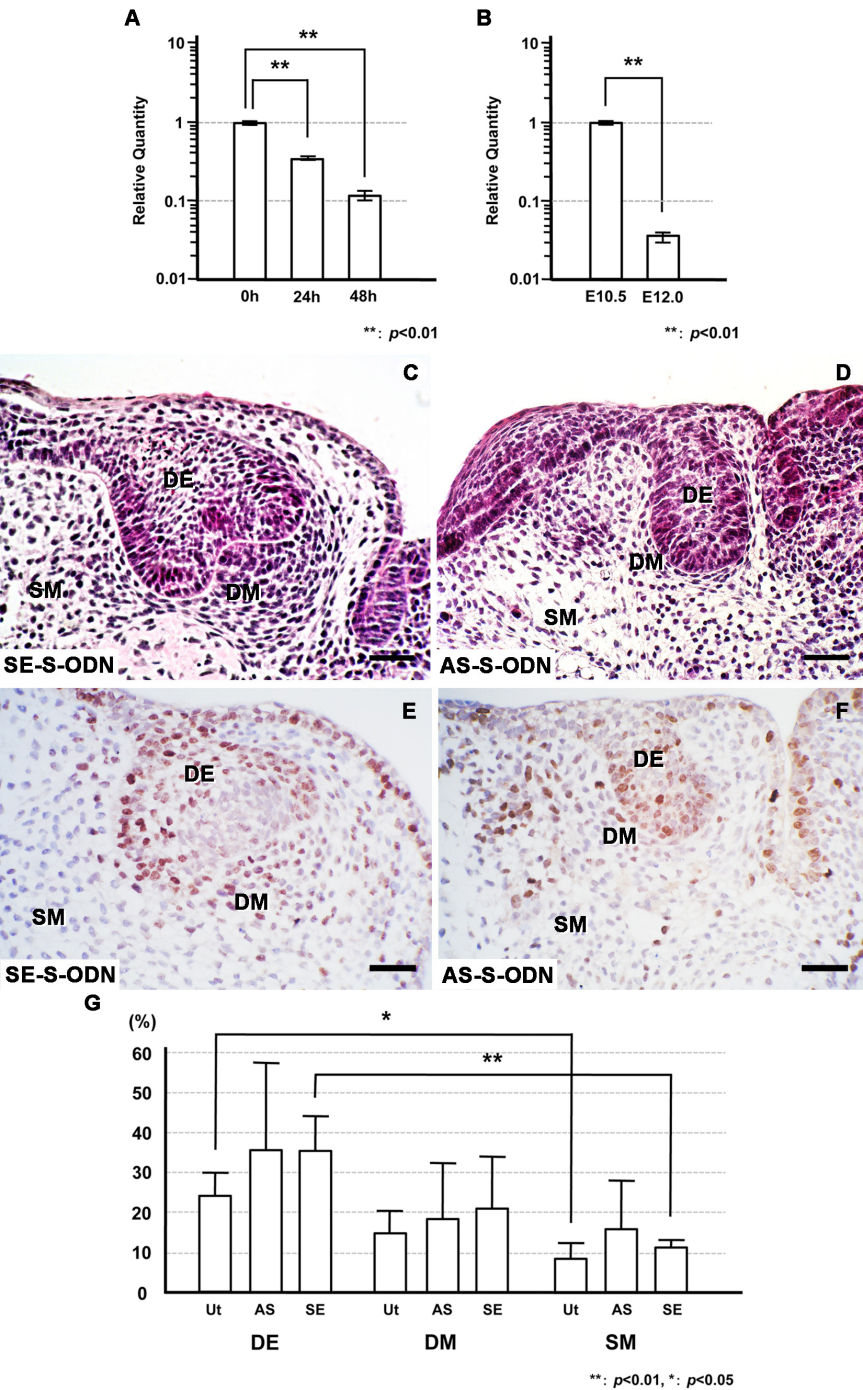
***Prtg *knockdown induces arrest of the tooth germ development**. (A) Prtg transcripts were dramatically decreased in the E10.5 mandible in a time-dependent manner in the organ culture (** *p *< 0.01). (B) Prtg expression in the E12.0 mandible was reduced to less than 5% of that in the E10.5 mandible (** *p *< 0.01). Tooth germ in the control sample or in the mandible treated with SE-S-ODN (C) developed to the cap stage on the 8th day of cultivation, while (D) an arrest of the tooth germ development was observed in samples treated with the AS-S-ODN. Immunohistochemically, Ki67-positive cells were seen in the control samples and in the mandibles treated with SE-S-ODN (E), and in samples treated with AS-S-ODN (F) on the 8th day after cultivation. (G) There were no significant differences in the cell proliferation in the objective areas among the control samples, the samples treated with AS-S-ODN and the samples treated with SE-S-ODN. Significant differences in the cell proliferation were noted between the DE and SM in the control samples and in the mandible treated with SE-S-ODN, while this was not observed among the areas in samples treated with AS-S-ODN (*p < 0.05, ** *p *< 0.01). The left sides of Figures 7C, D, E and F correspond to the buccal side, while the right side corresponds to the lingual side. Scale bars: C, D, E and F, 150 μm. Ut; untreated control sample, AS; samples treated with AS-S-ODN, SE; samples treated with SE-S-ODN. DE; dental epithelium, DM; dental mesenchyme, SM; surrounding mesenchyme (SM).

The expression of Prtg was time-dependently examined in the organ-cultured E10.5 mandibles because the expression level of Prtg markedly decreased after E12.0 in comparison to that at E10.5 (shown in the Figures 4A and 4C). The real-time PCR showed a marked decrease of the Prtg expression by 48 hr in mandible culture (Figure 7A) as well as that in E10.5 and E12.0 mandibles (Figure 7B). The Prtg expression in the mandibles cultured for 24 hr and 48 hr significantly decreased to less than 40% and 15% of that in the E10.5 mandible, respectively. The Prtg expression in the E12.0 mandible also showed a marked reduction to less than 5% of that in the E10.5 mandible.

A histological analysis was performed to evaluate the effects of *Prtg *knockdown on enamel organ formation in the cultured E10.5 mandible after phosphorothioated-oligodeoxynucleotide (S-ODN) treatment for 8 days. The period of organ culture was based on previous studies [6,7] in which the normal development of the tooth germ showed the cap stage by the 8th day of organ culture. As shown in Table 1, most of the cultured mandibles at E10.5 treated with Prtg AS-S-ODN showed an apparent inhibition of tooth germ development after being cultured for 8 days (Figure 7D and Table 1). In contrast, the mandibles treated with Prtg sense-S-ODN (SE-S-ODN) showed normal cap-like tooth germ (Figure 7C) as did the untreated mandibles and mandibles treated with only hemagglutinating virus of Japan (HVJ)-liposome (Table 1). The development of the enamel organs treated with Prtg AS-S-ODN on day 8 of culture was significantly inhibited in comparison to that in the other groups (*p *< 0.05; Table 1).

**Table 1:** The effects of Prtg knockdown on enamel organ formation in the cultured mandible.

Developmental Stages	Untreated		HVJ		SE		AS*	
				
	Bud	Cap	Bud	Cap	Bud	Cap	Bud	Cap
Sample No.	0	10	1	5	1	6	18	5
(%)	(0)	(100)	(17)	(83)	(14)	(86)	(78)	(22)

(*p < 0.05)

The histological analysis was performed to evaluate the effects of *Prtg *knockdown on enamel organ formation in the cultured E10.5 mandibles after S-ODN treatment for 8 days. The development of the enamel organs on day 8 of culture was significantly inhibited.

A cell proliferation analysis was performed to address the involvement of Prtg in the tooth morphogenesis. The Ki67-positive ratio was evaluated in the cultured organs treated with AS-S-ODN for Prtg. In the cultured organs, the objective cells were examined in three areas; the "dental epithelium (DE)", "dental mesenchyme (DM)" and "surrounding mesenchyme (SM)". The DM was either the "dental papilla and follicle" in the cultured organs showing the normal cap-like tooth germ, or the "odontogenic ectomesenchyme" in the samples with the inhibition of tooth germ development. As shown in Figure 7G, no significant difference in the Ki67-positive ratio was observed in any of the objective areas (DE, DM or SM) between the control mandibles (Figure 7E) and the cultured mandibles at E10.5 treated with Prtg AS-S-ODN (Figure 7F). While significant differences in cell proliferation were noted between the DE and SM in the control sampled and in the mandibles treated with SE-S-ODN, this difference was not observed in the areas in samples treated with AS-S-ODN (Figure 7G). No apparent inhibition of cell proliferation by Prtg perturbation was observed in the samples at day 8 of culture in this study.

### Down-regulation of Bmp-4 expression by the depletion of Prtg mRNA by AS-S-ODN

Based on the findings of the Prtg inhibition assay, we performed a real-time PCR analysis to examine *Bmp-4, Fgf8*, *Lef-1*, *Pitx2 *and *Shh *expression between AS-S-ODN-treated E10.5 mandibles and the others. These genes are expressed in mouse E10.5 mandible and are associated with odontogenesis [24-28]. The samples treated with S-ODN for 24 hr were used to examine the changes in gene expression in the early phase after Prtg inhibition.

**Figure 8:**
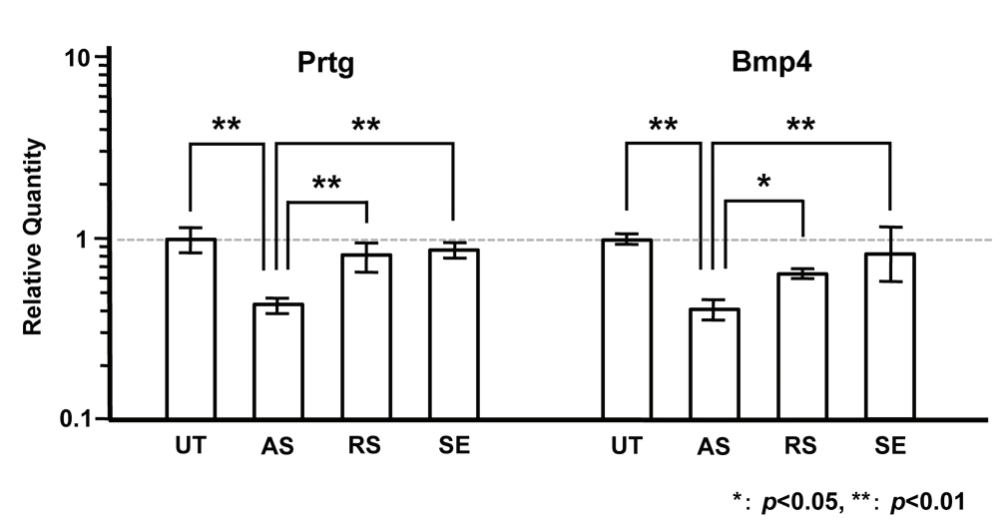
***Prtg *knockdown reduces the expression of odontogenesis-related genes**. Bmp-4 expression was significantly downregulated after 24 hr AS-S-ODN treatment in the cultured E10.5 mandibles. Significant differences in the transcription activity are indicated with either single or double asterisks (*p < 0.05, ** *p *< 0.01). Ut; untreated control sample, AS; samples treated with AS-S-ODN, RS; samples treated with random sequence-S-ODN, SE; samples treated with SE-S-ODN.

Mouse Gapdh was used as an internal control. At 24 hr after AS-S-ODN treatment, Bmp-4 mRNA expression in the E10.5 mandible was reduced to approximately 40% by AS-S-ODN treatment, and was significantly lower with AS-S-ODN treatment than without treatment, than with RS-S-ODN treatment, or with SE-S-ODN treatment samples (*p *< 0.05, *p *< 0.05 and *p *< 0.01, respectively; Figure 8). Meanwhile, the expression levels of *Fgf8, Lef-1, Pitx2*, and *Shh *showed no significant differences following treatment with Prtg AS-S-ODN (data not shown).

## Discussion

This study showed that *Prtg*, which is a highly expressed gene in the E10.5 mouse mandible, using a cDNA subtraction method between mandibles at E10.5 and E12.0 [9]. The gene belongs to the immunoglobulin superfamily according to a structural analysis, and was expressed in the early stages of the developing tooth germ. The temporal and spatial expression of this gene suggested that this gene is involved in the development of the mouse lower first molar. This is the first report to describe the relationship between the expression of *Prtg *and tooth morphogenesis.

The amino acid alignment of *Prtg *is comprised of an SP, 4 Ig domains, 5 FNIII repeats, a single TM, and a CD, thereby showing the typical structure of an IgSF. Treatment with N-glycosidase F revealed that Ptrg is a highly N-glycosylated transmembrane protein. However, because the Prtg protein is bigger than the assumed size even after the treatment with N-glycosidase, this modification is also associated with O-glycosylation and phosphorylation. The structural characteristics of Prtg are similar to DCC, Neogenin [17], Punc [18], and Nope [19], thus suggesting that they are all members of the DEAL subfamily.

Some IgSF proteins are important in the early developmental stage of the central nervous system. Meanwhile, these IgSF proteins are also implicated in various organs and tissues such as testis (BT-IgSF)[29], intestine (Neogenin) [17] and mesoderm cells (Robo)[30]. Chuong et al. [31] suggested that DCC may be involved in the differentiation of stem cells within several epithelial tissues. Nierhoff et al. [32] reported that the IgSF member *Nope *is expressed in rat fetal liver stem cells, and this gene might therefore be useful to identify, characterize, and isolate hepatic stem cells from the adult liver. Recently, diverse functions of DEAL have been identified, and include cell migration and axon growth guidance during development of the vertebrate nervous system [33-37] and the morphogenesis of epithelial tissues and the control of apoptosis in non-neural organization [17,31-33,38-40]. Therefore, it is likely that Prtg has multiple functions as a member of the DEAL family, including cell proliferation and cell differentiation during embryogenesis.

The RT-PCR and Western blot analyses showed that the Prtg mRNA and protein were expressed in the early developmental stages of tooth germ morphogenesis, as well as in the central nervous system. In the mouse, we did not detect Prtg mRNA in any adult tissues except for the brain, thus suggesting that Prtg function may be required in some non-neural tissues during organogenesis. The ISH and IHC results demonstrated that this gene was highly expressed in the epithelial and mesenchymal cells at E10.5, and this expression pattern is similar to the distribution of the arch ectodermal cells [14]. The first branchial arch is derived from neural crest cells [23]. *Ptrg *is strongly expressed in the first branchial arch in the early stage of embryogenesis (until E9.25) [14]. Because the mandible develops from the first branchial arch, the findings suggest that Prtg might play a role in the migration of neural crest cells, similar to the function of the other DEALs during development of the nervous system [33-37]. Tooth morphogenesis appears to begin with a signal of an epithelial-mesenchymal interaction [3-5]. The site where the tooth germ is likely to form is determined by the signal of mesenchymal cells which are derived from craniofacial neural crest cells [23]. The mesenchymal cells in the early phase (E10.5 and E12.0) of tooth germ formation were found to be positive for Prtg. This may indicate that the expression of Prtg is closely associated with epithelial-mesenchymal interactions.

An interesting finding in this study revealed that Prtg was expressed in epithelial cells, including the estimated tooth germ formation area at E12.0, the epithelial cells of the tooth bud at E14.0, and the inner enamel epithelium at E18.0. The DEAL proteins have been observed in non-neural systems and are involved in the morphogenesis of epithelial tissues and the control of apoptosis during organization. In fact, there are reports describing their role in the differentiation of the intestine epithelium [41,42], and in the budding and branching of lung alveoli [39]. Therefore, it is reasonable to consider that Prtg is also involved in the early development of odontogenic epithelial tissue. Once the tooth developmental process is initiated, then Prtg expression may be dramatically downregulated in these regions. In this study, AS-S-ODN was employed in an organ culture system to examine the functional roles of *Prtg *in the development of the tooth germ. The development of tooth germ was arrested at the bud stage when AS-S-ODN was added to the culture media. However, Prtg perturbation did not lead to any apparent inhibition of cell proliferation in this study. The reason for the developmental arrest of the tooth remains unknown at present. It is possible that the result may have been observed because the comparison was made between the tooth germs with different developmental stages at the 8th day after cultivation of the controls or the samples treated with SE-S-ODN and the samples treated with Prtg AS-S-ODN. Recently, Wong et al. reported that Prtg might have the potential to act before the onset of circulation to coordinate the rate of proliferation and the time of differentiation between the three primary germ layers [43]. In our study, the expression level of Prtg markedly decreased after E12.0 in comparison to that at E10.5. Similar results were shown during nerve development in the study of Wong et al. [43]. Therefore, it seems likely that the Prtg participates in the development of the tooth germ during the process of odontogenesis.

Furthermore, Bmp-4 mRNA expression was decreased following Prtg depletion. This result suggests that the Prtg is related to the direct or indirect regulation of Bmp-4 gene transcription. Because Bmp-4 plays an important role during odontogenesis as well as embryogenesis [24,26], Prtg depletion by treatment with AS-S-ODN may induce developmental arrest of the tooth germ. However, there have been no reports thus far that describe the mechanism of Prtg regulation of Bmp-4 expression. The other genes, Fgf8, Lef-1, Pitx2, and Shh, also play important roles in determining and/or budding tooth germ in the early development. Bmp-4 induces Lef-1 expression [44]. Pitx2 transcription is partially regulated by Lef1 [45]. Meanwhile, the *Lef-1 *expression overlaps that of *Pitx2 *at approximately E1.5 after *Pitx2 *expression, and Pitx2 regulates the Lef-1 isoform expression [46]. Interactions between these products are complex, and further studies will likely clarify the interrelationship between them and their role in development. Although Bmp-4 downregulation would be expected to extend to other genes, Prtg depletion did not significantly affect the expression of these genes in the early phase (within 24 hr). While *Pitx-2 *is expressed within the entire left atrial chamber of E12.5 mouse hearts [47], the Prtg protein is not detectable within cardiac cells of the atrial and ventricular chambers from E8.25 to E10.5 mouse hearts [43]. The interaction between Prtg and these proteins, and signal transduction pathways associated with Prtg have not been identified so far. Therefore, future studies will be needed to clarify the interaction between Prtg and Bmp-4 during signal transduction and subsequent gene expression, as well as that among the other genes during tooth germ development.

Alternatively spliced variants were not identified within the coding region of Prtg cDNA from E10.5 mice in this study. In contrast, mouse Neogenin (mNeogenin) has four alternatively spliced exons, three within the extracellular domain and a fourth within the cytoplasmic domain. Three of these alternatively spliced exons are developmentally regulated [48]. Interestingly, DCC also contains an alternatively spliced exon within the extracellular domain [49,50]. The expression of DCC with this alternative exon is also regulated throughout embryogenesis, as seen with the alternative forms of mNeogenin [51]. Thereafter, when more clones from various embryonic stages are analyzed, alternatively spliced forms of Prtg may be found in the neuron tissue. However, Prtg expression was dramatically downregulated in the tooth germ after E12. The regulation of Prtg function might therefore be different from the mechanism of DCC and Neogenin.

## Conclusion

This study demonstrated that Ptrg is preferentially expressed in the early stage of organogenesis. This study characterized the expression pattern of *Prtg *in the developing tooth germ, and thus shows the possible functional implications of this gene in tooth germ morphogenesis through an inhibition assay for Prtg by AS-S-ODN in organ culture. Prtg, an IgSF family member is involved in the initial development of the tooth germ and in the differentiation of the inner enamel epithelial cells in the mouse lower first molar. Future investigations of organ cultures of the mandible earlier than E10.5 are therefore expected to clarify this process.

## Methods

### Animals

The embryos of BALB/c mice at E10.5, E12.0, E14.0, E16.0, and E18.0 after gestation were used in this study. The adult BALB/c mice were obtained from Charles River Laboratories (Charles River Japan Incorporated). Female BALB/c mice (10-30 weeks) were caged together with male mice. After 3 hr, successful insemination was determined based on the presence of a post-copulatory plug in the vagina. The embryonic day was defined as E0 after the post-copulatory plug was recognized. Male mice (3, 5, and 10 weeks) were also used to examine the expression of Prtg. All mouse experiments and housing were performed in accordance with the guidelines of the Animal Center of Kyushu University.

### cDNA subtraction and cloning procedures

*Prtg *was identified as a novel gene, termed *Clone 15*, in a previous study [9]. Based on the sequence of a fragment of this gene, 5'-/3'-RACE was performed to determine the full-length sequence (SMART RACE cDNA Amplification Kit; Clontech). *Prtg *DNA sequencing was performed with the dideoxynucleotide termination method using a DNA sequencer 373 S (Applied Biosystems). A search of the GenBank online database (using NCBI/BLAST/blastn suite: BLASTN programs) revealed no information at that time. In a later search, it corresponded with part of GenBank Accession numbers AK036172, AK083540, and NM_175485. AK036172 includes a polyadenylation signal site, and NM_175485 includes an arrangement of a signal peptide (SP). The sequencing data identically correlated to that of the recently updated NM_175485.4.

### Structural analysis based on the amino acid alignment

A domain analysis of the Prtg protein sequence was carried out based on the amino acid alignment using an NCBI conserved domain search with the online NCBI program http://www.ncbi.nlm.nih.gov/Structure/cdd/cdd.shtml. The signal peptides and a transmembrane region were predicted using the SOSUI system http://bp.nuap.nagoya-u.ac.jp/sosui/.

### Intracellular localization of recombinant Prtg

Three different plasmids expressing an EGFP-fusion protein were prepared. Prtg cDNA with a full-length, or the Prtg cDNA with a deleted SP1 or SP2 region were inserted in pEGFP-N1 vectors (Clontech), as shown in Figure 2A. These subcloned vectors were termed as Prtg-full, Ptrg-ΔSP1, and Prtg-ΔSP2. MISK81-5, which is an oral squamous cell carcinoma cell line established in our laboratory [22], was stably transfected with Prtg-full, Prtg-ΔSP1, Prtg-ΔSP2, or an empty vector using Lipofectamine 2000 (Invitrogen). These transfectants were isolated after selection with 800 μl/ml G418 for 2-3 weeks.

Immunofluorescent staining with an anti-cadherin antibody and Alexa Fluor 594 rabbit anti-mouse IgG (Invitrogen) was performed on the cells transfected with the Prtg-full plasmid. The fluorescent images were observed under a fluorescent microscope and acquired using the digital imaging software program, AxioVision version 3.1 (Carl Zeiss).

### Specific antibodies against Ptrg

Rabbit anti-Prtg polyclonal antibodies were generated against a synthetic peptide based on the regions as follows: 1) PKDASESNQRPKRLDSSNAKV (Entrez Protein database accession number NP_780694 aa 910-930), 2) STPPTSNPLAGGDSDGDAAPKKHGD (aa 1139-1163), and 3) DAAPKKHGDPAQPLPA (aa 1156-1171). The first amino acid sequence is present in the extracellular domain near the transmembrane region, whereas the two latter sequences correspond to sequences in the cytoplasmic domain. First, three antibodies were tested for their dye-affinity. The antibody for aa 1156-1171 was selected and used for all subsequent experiments because it gave a more specific signal.

### Western blot analysis

A Western blot analysis for Prtg protein levels was performed on resolved proteins isolated from the homogenates of E10.5 and E12.0 mandibles and E18.0 tooth germ. These tissues were lysed in RIPA buffer (50 mM Tris pH 8.0, 150 mM NaCl, 1% Triton X-100, 1 mM EDTA pH 8.0, 0.1% SDS) supplemented with a protease inhibitor cocktail (50 μM), lactacystin (20 μM), and PMSF. The protein samples were separated on a 7% SDS-polyacrylamide gel and electrotransferred to an Immun-Blot PVDF Membrane (Bio-Rad). The membrane was probed with antibody against Prtg for 1 hr at room temperature, and incubated for 1 hr with secondary anti-rabbit IgG conjugated with horseradish peroxidase (Amersham). The membrane was developed using the enhanced chemiluminescence (ECL) Plus system (Amersham). Emitted light was detected using a cooled CCD-camera (LAS-1000), (Fujifilm). Glycosidase digestion was performed with an N-glycosidase F deglycosylation kit (Roche) according to the manufacturer's instructions before loading the samples in the gel.

### Temporal expression analysis of Prtg mRNA by semi-quantitative RT-PCR

Total mRNA was extracted from E10.5, E14.0, and E18.0 mice, and from various organs of the 3-, 5-, or 10-week-old mice using an SV Total RNA Isolation system (Promega). Reverse transcription was performed to synthesize cDNAs using the Superscript III reverse transcriptase (Invitrogen). The cDNAs were amplified by PCR to compare the expression with the manifestation quantity. The forward and reverse primer pairs for Prtg and glyceraldehyde-3-phosphate dehydrogenase (GAPDH) were: Prtg 5'-CGA AGC AAA GCC AGG AAG TC-3' and 5'-GCT TGT TGT GAA TCC CTG AGC G-3', and GAPDH 5'-ACC ACA GTC CAT GCC ATC AC-3' and 5'-TCC ACC ACC CTG TTG CTG TA-3'. The PCR products were separated by electrophoresis on a 2% agarose gel. To confirm that the PCR products were derived from cDNA and not from genomic DNA, the primer pairs were designed upstream and downstream of introns.

### *In Situ *Hybridization

The section preparation, the probe labeling, the specificity of the DIG-labeled *in situ *RNA probes, and ISH methods were carried out as described in our previous studies [6,7,12]. *Prtg *antisense probes were designed against a sequence in the C-terminal region and the 3'-UTR corresponding to nucleotide positions 3487 to 4917 (NM_175485.4). A *Prtg *sense probe was applied to the tissue specimens as a control. However, no hybridization signal was detected.

### Immunohistochemistry

The preparation of serial cryosections was processed in the same way as for ISH. After the dried cryosections were rinsed with PBS containing 0.1% Triton X-100 for 10 min, and IHC was performed with a CSA II Biotin-free Tyramide Signal Amplification System according to the manufacturer's instructions (Dako). The primary anti-Prtg antibody diluted 1:500 in PBS was used. For the negative control, the application of the primary antibody was omitted from the procedure.

### Inhibition assay for Prtg by AS-S-ODN in organ culture

The detailed procedures of the inhibition assay for Prtg by AS-S-ODN in organ culture have been shown in previous studies [6,7]. Briefly, the mandibles were dissected from E10.5 embryos. These explants were mounted on a filter (0.8 μm pore size, Millipore, MA), and then were incubated in Fitton-Jackson's modified BGJb medium (Invitrogen) supplemented with 5% fetal bovine serum (Filtron, Brooklyn, Australia), 100 μg/ml ascorbic acid (Invitrogen), and 100 units/ml penicillin/streptomycin (Invitrogen) in a 5% CO2 atmosphere at 37°C [6,7].

The HVJ-liposomes (GenomeOne series, Ishihara Sangyo Kaisha, LTD., Osaka, Japan) were purchased for this study. The HVJ-liposome complex was prepared according to the manufacturer's instructions (Ishihara). ODNs were: sense-S-ODN (SE-S-ODN): 5'-TGA ATG GCG CCT CCC GT-3', antisense-S-ODN (AS-S-ODN): 5'-ACG GGA GGC GCC ATT CA-3'. The SE/AS-S-ODNs corresponded to nucleotide positions 181-197 (GenBank accession number NM_175485.4). The treatment with AS- or SE-S-ODN or HVJ-liposome alone was performed at 24 hr intervals.

### Histological analysis of cultured mandibles

On the 8th day after cultivation, the cultured mandibles were fixed with 4% paraformaldehyde and embedded in paraffin to be used for histological analysis. Five-μm-thick sections were cut in an antero-posterior direction, and were stained in hematoxylin and eosin. The sections were examined under light microscopy.

### Cell proliferation analysis of the cultured organs treated with AS-S-ODN

In order to address the involvement of the Prtg protein in tooth morphogenesis, a cell proliferation was analyzed in the cultured organs treated with AS-S-ODN for Prtg. Immunohistochemistry using a rabbit polyclonal antibody to the Ki67 (Abcam, Cambridge UK) was performed to evaluate Ki67-positive cells in the DE, DM and SM areas. More than one hundred objective cells were examined as a population in at least three different microscopic fields of each area. The number of the stained cells was divided by the total number of stained and non-stained target cells to calculate the Ki67-positive ratio.

### Effects of Prtg suppression by AS-S-ODN on odontogenesis-related gene transcription

Real-time quantitative PCR was performed to estimate the subsequent expression of selected genes using Thermal Cycler Dice Real Time System (TaKaRa, Shiga, Japan) with SYBR Premix Ex Taq II (TAKARA) according to the manufacturer's instructions. At 24 hr after the inhibition assay for Prtg by AS-S-ODN in organ culture, *Bmp-4, Fgf8*, *Lef-1*, *Pitx2*, and *Shh *were analyzed in this study. Gapdh was used as a referable gene. The specific primer sets were as follows:

Prtg forward 5'-ATC GCA GTA GGC GTT GGC ATA-3',

reverse 5'-CGC TGT CTT AGA GGC GGA TGA-3'.

Bmp-4 forward 5'-AGC CGA GCC AAC ACT GTG AG-3',

reverse 5'-TCA CTG GTC CCT GGG ATG TTC-3'.

Fgf8 forward 5'-CAT CAA CGC CAT GGC AGA A-3',

reverse 5'-TCT CCA GCA CGA TCT CTG TGA ATA C-3'.

Lef1 forward 5'-TCA CTG TCA GGC GAC ACT TC-3',

reverse 5'-TGA GGC TTC ACG TGC ATT AG-3'.

Pitx2 forward 5'-AGC TGT GCA AGA ATG GCT TT-3',

reverse 5'-CAC CAT GCT GGA CGA CAT AC-3'.

Shh forward 5'-AGC AGA CCG GCT GAT GAC TC-3',

reverse 5'-TCA CTC CAG GCC ACT GGT TC-3'.

Gapdh forward 5'-TGT GTC CGT CGT GGA TCT GA-3',

reverse 5'-TTG CTG TTG AAG TCG CAG GAG-3'.

The relative expression levels of each targeted gene were normalized using the ΔΔC_T _comparative method, based on the referable gene threshold cycle (CT) values [52].

### Statistical analysis

Significant differences within a group and between groups were determined by a chi-square test for the independence of the Prtg inhibition assay, and by unpaired Student's t-test or one-way ANOVA with the Tukey-Kramer comparison test for real-time PCR data and cell proliferation assay using the Statcel2 software program. When necessary, Welch's t-test was used for unequal variances. A *P-*value of less than 0.05 or 0.01 was considered to be statistically significant.

## Authors' contributions

KFT and TK carried out the experimental work. KFT performed the immunoassays, *in situ *hybridization and the organ culture studies. KFT also helped to draft the manuscript. TK carried out the molecular genetic studies, and performed the statistical analysis. TK also participated in the design of the study and coordination, and helped to draft the manuscript. IK, MX, HF, YO and HW helped to conduct the organ culture studies with AS-S-ODN, and helped to make the histological analysis. HY participated in the sequence alignment and structure analysis. IK, KN and HW participated in the histological analysis and cell proliferation analysis. TS and YT helped to review the data. HS conceived the study, participated in its design and coordination, and drafted the manuscript. All authors read and approved the final manuscript.
